# Genetic Factors of Cerebral Small Vessel Disease and Their Potential Clinical Outcome

**DOI:** 10.3390/ijms20174298

**Published:** 2019-09-03

**Authors:** Vo Van Giau, Eva Bagyinszky, Young Chul Youn, Seong Soo A. An, Sang Yun Kim

**Affiliations:** 1 Department of Bionano Technology & Gachon Bionano Research Institute, Gachon University, Seongnam-si, Gyeonggi-do 461-701, Korea; 2 Department of Neurology, Chung-Ang University College of Medicine, Seoul 06973, Korea; 3 Department of Neurology, Seoul National University College of Medicine & Neurocognitive Behavior Center, Seoul National University Bundang Hospital, Seoul 06973, Korea

**Keywords:** CADASIL, CARASIL, CARASAL, SVD, HDLS, ischemic stroke, vascular dementia, young-onset stroke, genetic molecular analysis

## Abstract

Cerebral small vessel diseases (SVD) have been causally correlated with ischemic strokes, leading to cognitive decline and vascular dementia. Neuroimaging and molecular genetic tests could improve diagnostic accuracy in patients with potential SVD. Several types of monogenic, hereditary cerebral SVD have been identified: cerebral autosomal recessive arteriopathy with subcortical infarcts and leukoencephalopathy (CARASIL), cerebral autosomal-dominant arteriopathy with subcortical infarcts and leukoencephalopathy (CADASIL), cathepsin A-related arteriopathy with strokes and leukoencephalopathy (CARASAL), hereditary diffuse leukoencephalopathy with spheroids (HDLS), *COL4A1/2*-related disorders, and Fabry disease. These disorders can be distinguished based on their genetics, pathological and imaging findings, clinical manifestation, and diagnosis. Genetic studies of sporadic cerebral SVD have demonstrated a high degree of heritability, particularly among patients with young-onset stroke. Common genetic variants in monogenic disease may contribute to pathological progress in several cerebral SVD subtypes, revealing distinct genetic mechanisms in different subtype of SVD. Hence, genetic molecular analysis should be used as the final gold standard of diagnosis. The purpose of this review was to summarize the recent discoveries made surrounding the genetics of cerebral SVD and their clinical significance, to provide new insights into the pathogenesis of cerebral SVD, and to highlight the possible convergence of disease mechanisms in monogenic and sporadic cerebral SVD.

## 1. Introduction

**Figure 1:**
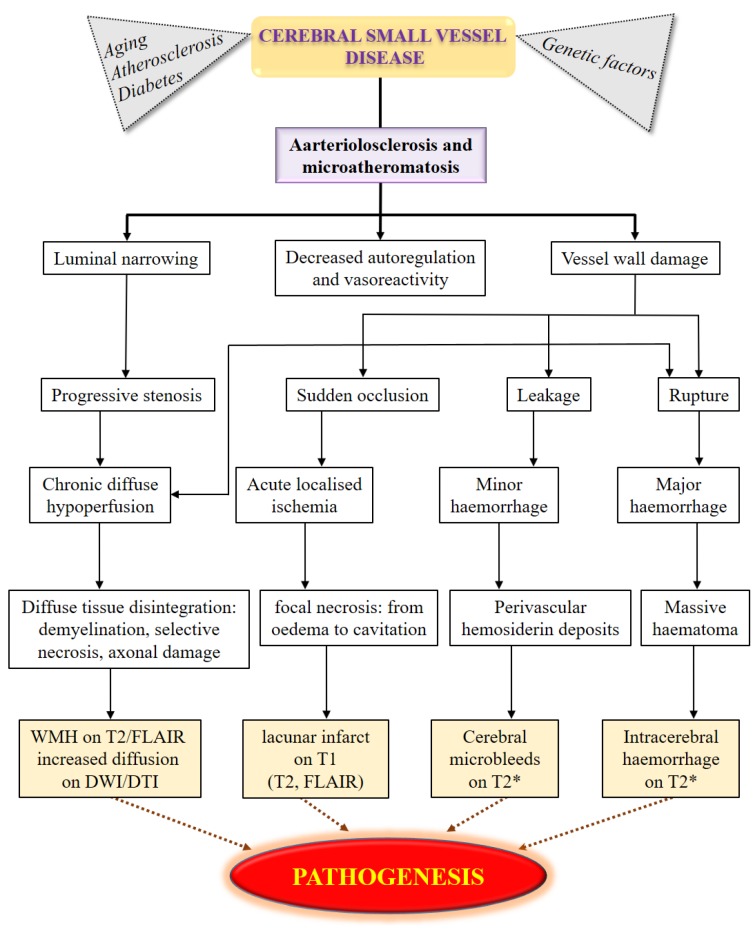
Pathogenesis of cerebral SVD manifestations in development and its progression.

Cerebral small vessel diseases (SVD) has been recognized as an important cause of cognitive impairment and dementia among the elderly [1,2,3]. The pathologic correlation of cerebral dysfunctions with stroke was stronger in the population in Asia than in the USA or Europe [1]. Cerebral SVD accounts for 15–26% of ischemic strokes in the USA and Europe [4,5,6,7], whereas this proportion in Asia ranges from 25% to 54% [8,9,10,11,12]. However, these observations were made only in hospital-based settings, and the studies were performed in non-Asian populations [13,14]. Cerebral SVD has an enormous social and economic impact. Currently, the causes of the majority of cerebral SVD are not well understood, which could limit effective treatment. Several traditional risk factors have been suggested to play important roles in the mechanisms and etiology of cerebral SVD [15]. In addition, a comparative investigation of vascular risk factors, as well as genetic and environmental susceptibility has suggested that ethnic differences could contribute to the prevalence of cerebral SVD. Generally, cerebral SVD subtypes may present similar pathogenesis, comprising lacunar stroke, hypertensive hemorrhage, leukoaraiosis, or cerebral microbleeds [16,17,18], which indicate a possible overlapping etiology with extracerebral small vessel vasculopathies [19,20,21,22]. The standard markers of SVD included lacunes, cerebral microbleeds, or white matter hyperintensities (WMHs). These markers, particularly in advanced structural neuroimaging magnetic resonance imaging (MRI) techniques, were used to assess the accumulated burden of cerebrovascular disease throughout life. However, the variations in the definitions and terms of descriptive features may cause difficulties with respect to the interpretation and comparison of results between studies. Until recently, the molecular, cellular, and pathophysiologic mechanisms underlying SVD were largely unknown. Mechanistic studies were hampered by a lack of animal models, difficulties in visualizing small blood vessels in vivo, particularly with technological challenges in making an animal model of brain microvessels for the physiologic and biochemical studies. Figure 1 summarized the pathogenesis of cerebral SVD manifestations. Although rare, these phenotypic extremes shared both clinical and radiological features with sporadic SVD, which could provide important insights into the mechanisms of the disease. Thus, diagnostic and therapeutic strategies are still limited [1,2,3]. 

Genetic disorders could be based on the combination of mutations in a single gene or on a cluster of genes with an autosomal dominant or recessive pattern [2,23,24]. Interestingly, particular traits suggested recessive patterns by receiving chromosomes from both parents, where different mutations of identical genes were responsible [21]. Similar to ischemic stroke, genetic factors revealed their significant impact on cerebral SVD, which could clarify the pathophysiology of sporadic cerebral SVD. The estimated heritability for cerebral white matter lesions as a surrogate marker of cerebral SVD ranged between 50% and 80% [25]. Since the human genome project in 2003, extraordinary progress has been made in genome sequencing technologies, genome-wide association studies (GWAS), and the meta-analysis of individual candidate gene studies. These advancements have contributed to a significant increase in the number of, and in-depth information available on the genetic loci for sporadic cerebral SVD [1]. Since the occurrences of cerebral SVD would increase in all age groups, in-depth investigations on the molecular mechanisms of the pathogenesis of cerebral SVD were needed [26]. Although the majority of cerebral SVD cases were sporadic, several reports of inherited forms of cerebral SVD pointed to single-gene disorders. Different monogenic cerebral SVD were discovered (Table 1), including cerebral autosomal-dominant arteriopathy with subcortical infarcts and leukoencephalopathy (CADASIL) [27], cerebral autosomal recessive arteriopathy with subcortical infarcts and leukoencephalopathy (CARASIL) [28,29], cathepsin A–related arteriopathy with strokes and leukoencephalopathy (CARASAL) [30], hereditary diffuse leukoencephalopathy with spheroids (HDLS) [31], COL4A1/2-related cerebral SVD [27,32,33], autosomal-dominant retinal vasculopathy with cerebral leukodystrophy, and Fabry disease. Thus, the recognition of the genetic aspect of cerebral SVD could contribute to the improved diagnosis and treatment of these rare single-gene disorders, as well as sporadic cerebral SVD. 

The clinical features of autosomal-dominant SVD may share several similarities with sporadic SVD, such as arteriopathy, white matter dysfunctions, and infracts in subcortical regions [1,34,35]. These diseases could have wide a wide range of age of onset—some patients develop SVD at a younger age (under 20 years), while in other affected individuals the disease phenotypes could present after age 50 or 60 [26,35]. Genetics could play a significant role in the onset of SVD. Thus, genetic testing as part of routine diagnosis could identify the potential causative mutations in patients with a family history, suggesting possible connections between gene mutations and clinical symptoms of blood vessel diseases. The present work aimed to discuss the genetic background of the various forms of inherited cerebral SVD and the recent advances made in the genetic study of cerebral SVD pathogenesis.

## 2. Cerebral Autosomal-Dominant Arteriopathy with Subcortical Infarcts and Leukoencephalopathy (CADASIL)

**Figure 2:**
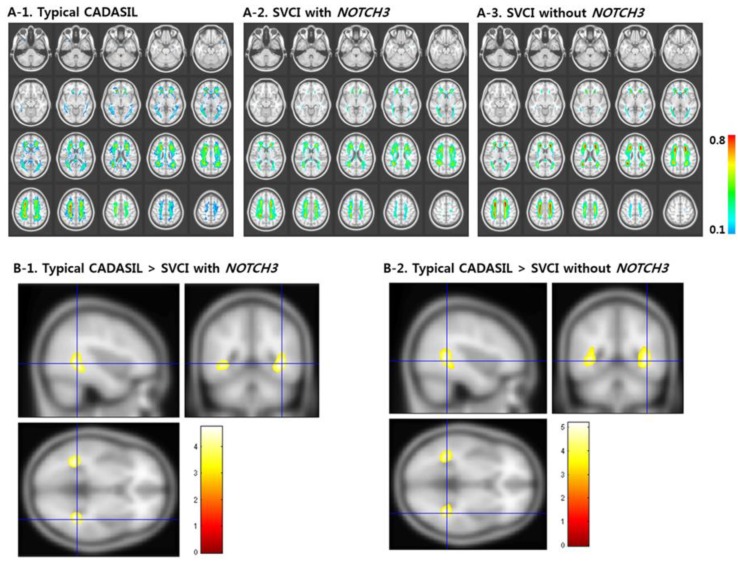
Multimodal imaging analyses in CADASIL and SVCI patients with and without NOTCH3 variants. WMH frequency maps of forms of CADASIL (**A**) and comparison of frequency maps between the typical CADASIL and SVCI groups (**B**). (**A**-**1**) Typical CADASIL patients show extensive WMH distributed throughout the periventricle, posterior temporal white matter, and anterior temporal white matter. (**A**-**2**) SVCI patients with NOTCH3 variant and (**A**-**3**) SVCI patients without NOTCH3 variants showed similar WMH frequency maps. (**B**-**1,B**-**2**) Typical CADASIL cases reveal significantly prevalent WMH distribution in the bilateral posterior temporal region compared with SVCI patients with and without NOTCH3 variants. Reprinted with permission from ref [40]. Copyright under a CC BY license (Creative Commons Attribution 4.0 International License).

CADASIL (OMIM 125310) is the most common hereditary stroke disorder, and it was also suggested as the most common inherited form of vascular dementia. Clinical symptoms appear at younger ages or during the adulthood. Common symptoms at the initial stage of disorder were stroke, migraine (with or without aura), or transient ischemic attack. Additional symptoms include cognitive impairment, dementia, mood disturbances or seizures [36]. CADASIL was associated with non-amyloid and/or non-atherosclerotic angiopathy in the brain vessels (small penetrating and lepto-meningeal arteries). The disease also affected the vessels in other organs, such as the muscles, skin, or heart [37]. One of the causes in ischemic brain injury was microangiopathy with granular osmiophilic deposits (GOM) in the basal membrane. The diagnosis of GOM could be confirmed with 100% accuracy using magnetic resonance imaging (MRI). Recently, skin biopsies were suggested [38]. MRI and computed tomography scan (CT) could reveal the hyperintensities and hypodensities in the white matter, respectively. Additionally, lacunar infarcts could be seen in different parts of the brain, such as the semioval center, thalamus, and basal ganglia. The third type of lesions were the cerebral microbleeds (CMBs) [39]. Affected brain areas were the thalamus, central semivovale, basal ganglia, temporal lobes or pons [37,40]. The imaging data of a typical CADASIL (A1), the atypical form of CADASIL with (A2) and without genetic mutations (A3), were summarized in Figure 2. Typical CADASIL manifests extensive white matter hyperintensities (WMHs) in different regions, which were less prominent in the atypical form. Patients with atypical CADASIL had stronger microstructural alterations in the bilateral frontal and temporal lobes and corpus callosum. The manifestation of similar symptoms and/or findings in relatives would strongly suggest familial CADASIL. Davous first suggested the diagnostic criteria for CADASIL in 1998 [41]. The clinical diagnosis was usually made on the basis of a combination of otherwise unexplained cerebral ischemic events or cognitive impairment, brain MRI abnormalities [42,43], and a family history of stroke or dementia [44]. 

Genetically, the missense mutations of cysteine-altering in *NOTCH3* gene on chromosome 19 [27] with 33 exons were responsible for CADASIL. The *NOTCH3* gene encoded for the 2321-amino-acid-long single-pass transmembrane receptor protein, one of the key moleculea of notch signaling, particularly during embryonal development [45]. In adults, NOTCH3 could be essential for the development, remodeling and differentiation of vascular system. A recent study revealed that patients with typical CADASIL with *NOTCH3* variants showed distinct anatomic vulnerabilities in both grey and white matter structures [40] (Figure 2).

The extracellular domain of NOTCH3 (NOTCH3^ECD^) consisted of 34 epidermal growths factor-like repeats (EGFr). These EGFrs subunits of approximately 40 amino acids contained three disulfide bridges in their positions, stabilizing their domain structures. The unpaired cysteine residue from the mutation in NOTCH3^ECD^ disrupted the disulfide bridge formation, and the shuffling and mismatched disulfide bridges would result in increased instability and multimerization properties in comparison to those in the wild-type NOTCH3^ECD^ [46], destroying the NOTCH3 signaling cascade. Hence, these multimerized toxic aggregations of NOTCH3^ECD^ were found in and around vascular smooth muscle cells (VSMCs) of small-to-medium-sized arteries, particularly in the brain. In addition, the multimeric depositions, which were also found in arteries of other organs and tissues [47,48]. The NOTCH3^ECD^ aggregation could also reflect the pathological changes in the vessel wall, including the degeneration of VSMCs and the deposition of visualized granular osmiophilic materials by electron microscopy [49].

The majority of pathogenic mutations in *NOTCH3* were missense mutations with rare deletions and insertions in one of 34 EGFr domains [50]. Although various *NOTCH3* mutations have been reported in different populations, all CADASIL mutations occurred in exons 2–24, resulting in a gain or loss of cysteine residues in the extracellular N-terminal region [51], creating an uneven number of cysteine—either five or seven—which commonly induced CADASIL. However, several reports have described non-cysteine related mutations [52], referring to these as non-classical *NOTCH3* mutations of CADASIL-like GOM-negative familial phenotypes with vascular dementia. Thus, clinical examination may raise suspicions for CADASIL, and the diagnosis should be confirmed by genetic testing, or by assessing for the detailed pathological characteristics of the disease (Table 2).

The recommendations for clinical diagnosis and interpretation of *NOTCH3* (Notch homolog 3, Drosophila) mutations in CADASIL were suggested by Rutten et al. [44]. In general, a genetic test presenting an archetypal CADASIL mutation [27] of *NOTCH3* gene would confirm the diagnosis [53]. If a mutation was found previously in an affected relative, the targeted molecular genetic testing could be performed for the particular loci. Otherwise, the whole gene or exons 2–24 (which harbor the majority of the mutations) should be sequenced [50]. The correct diagnostic interpretation of variants other than the stereotypical cysteine-altering missense mutations would require an expert opinion based on both the clinical features and distinguishing molecular aspects of CADASIL [44]. A recent study suggested that a single-particle in vitro aggregation assay might be a reliable tool to evaluate the clinical significance of the non-cysteine variants [54]. However, the study included only one family, and it was still debated whether the diagnosis was conclusive for selecting the method as diagnostic tool [44,54]. To date, more than 130 different mutations in *NOTCH3* gene were reported in patients with CADASIL, 95% of which were missense point mutations [51]. CADASIL is most commonly a hereditary autosomal-dominant disease affecting all small cerebral arteries. In addition, recent studies of five patients with homozygous mutations (p.Arg133Cys, p.Arg578Cys, p.Gly528Cys, p.Arg544Cys, and p.Cys183Ser) supported their pathological effects [55,56,57,58,59]. Interestingly, several cases of de novo mutations were also found [47,60,61].

Five small deletions (four in non-frame [47,62,63] and one frameshift), one splice site mutation [64], and a small deletion of a non-cysteine related residue were reported [52]. Further, p.Ala1020Pro, p.Arg213Lys, p.Tyr1098Ser, and p.Arg75Pro variants were found in diagnosed patients with CADASIL (Table 2) [65,66,67,68,69,70,71,72,73,74,75,76,77,78], in whom exons 2–24 were sequenced from skin biopsy for confirmation [79,80,81,82]. Recently, a three-nucleotide insertion was reported as the first pathogenic insertion in the *NOTCH3* gene [83]. However, the significance of such molecular variants still remains unclear. Therefore, complete screening of Notch3-coding exons should be performed in suspected cases for understanding the CADASIL phenotype and genotype spectra.

## 3. Cerebral Autosomal Recessive Arteriopathy with Subcortical Infarcts and Leukoencephalopathy (CARASIL)

A variety of rare genetic disorders may have symptoms similar to those found in CADASIL [67]. CARASIL was the second known form of ischemic, non-hypertensive, cerebral SVD with an identified gene, *HTRA1* [28,86,87,88]. Patients would develop several phenotypes in the early lifetime (teenage years or 20–30 years old), such as lumbago, alopecia or even encephalopathy [72,73]. Additional symptoms may occur in patients older than 30 or 40 years of age, such as gait disturbances, premature scalp alopecia, ischemic stroke, acute mid- to lower-back pain, pseudobulbar palsy, pyramidal-or extrapyramidal symptoms, Babinski signs and progressive cognitive disturbances, leading to severe dementia [73,84]. Brain CT imaging revealed diffuse homogenous changes in white matter and dilatation in different areas, such as cerebral sulci. MRI imaging revealed WMH lesions, which could be the common characteristics of CARASIL [72,84,88]. Additional signs included multiple lacunar infarctions or cerebral angiography. Lesions could initially appear in the subcortical deep white matter, but later they appear in other brain areas such as the thalamus, cerebellum or brainstem [87,88]. Anterior temporal lobes and external capsules were affected, suggesting that CARASIL and CADASIL may share phenotypical similarities [87,89]. Unlike CADASIL, CARASIL may demonstrate no pathognomonic histological features; the histological findings most closely resemble those of nonhereditary ischemic cerebral SVD, or “earthen pipe phenomenon” [90]. A fibrous proliferation of the intima, hyaline degeneration of the media, loss of VSMCs, and thickening and fragmentation of the internal elastic lamina were observed in patients with CARASIL [88]. Granular appearance of GOMs were never observed in CARASIL [88,89]. These findings were limited to the cerebral small arteries—a skin biopsy was not helpful for the diagnosis [86,87].

CARASIL is a rare genetic disorder characterized by mutations in the *HTRA1* gene located on chromosome 10q (10q25.3-q26.2). It is a very rare autosomal recessive form of familial SVD. CARASIL was identified to have an autosomal recessive inheritance pattern. Patients with mutations tend to produce protein products with a low protease activity; these proteins were unable to repress signaling by transforming growth factor-β (TGF-β) [28]. Approximately 56 cases have been reported: 48 from Japan, eight from China [91,92,93], and one case each from Spain [94], Romania [95], Turkey [96], and America [97]. These cases are summarized in Table 3. The mutation could result in the loss of HTRA1 protease activity, leading to cerebral small-vessel arteriopathy. Recently, heterozygous *HTRA1* mutations have been described in patients with late-onset familial SVD [29,85,93,98,99,100]. It has also been speculated that the upregulation of TGF-β family signaling by the mutant *HTRA1* may be responsible for the non-neurological manifestations of CARASIL, for example, alopecia and degenerative spine disease [28].

To date, at least 16 mutations in the *HTRA1* gene have been identified in 25 families. These mutations were mostly located in exons 3–6 and may result in a decreased level of protease activity, leading to an increase in TGF-β signaling [101], which in turn could cause degeneration of smooth muscle cells in the cerebral small vessels and angiopathy. The majority of missense mutations have been reported among Japanese patients; in particular, four pathogenic homozygous mutations including two missense (p.Ala252Thr; p.Val297Met) and two nonsense (p.Arg302X; p.Arg370X) mutations were discovered in this cohort. These mutations resulted in reduction of the protease activity by 21–50%, which was not enough to repress by the TGF-β family. Conversely, the other nonsense mutation (p.Arg370X) caused the loss of HTRA1 protease activity by the nonsense-mediated decay of mRNA [28]. The diagnosis of CARASIL was confirmed through molecular genetic testing, which identifies characteristic mutations in the *HTRA1* gene. In CARASIL patients, mutations in the *HTRA1* gene may cause disturbances in the regulation of TGF-β signaling. This abnormally increased TGF-β signaling altered the small blood vessel structure in the brain [28]. These blood vessel abnormalities (described as arteriopathy) increased the risk of stroke highly, leading to neuronal loss in several areas of the brain. The dysregulation of TGF-β signaling may also underlie the hair loss and back pain in CARASIL patients, although the relationship between abnormal TGF-β signaling and these features have not yet been fully clarified. Abnormally increased TGF-β signaling seemed to cause the degeneration of VSMCs, because TGF-β played an important role in the differentiation of these cells.

## 4. Cathepsin A–Related Arteriopathy with Strokes and Leukoencephalopathy (CARASAL)

CARASAL is a novel hereditary adult-onset cerebral SVD. The disease was characterized by therapy-resistant hypertension, strokes, and slow and late cognitive deteriorations. Impairment in the lower cranial nerve function was also prominent, resulting in several symptoms, such as vertigo, motor dysfunctions (including facial nerves), refractory hypertension, as well as a dry mouth and eyes [30]. In CARASAL, the MRI pattern showed a diffuse, progressive leukoencephalopathy that preceded the onset of strokes and was disproportionate to the degree of clinical severity. There were also multifocal signal changes in the cerebral white matter and basal nuclei, thalami, and brainstem, a pattern suggestive of SVD [15]. These changes caused the leukoencephalopathy to become virtually diffuse. Hervé et al. reported a French family with autosomal-dominant vascular leukoencephalopathy with an MRI pattern similar to that in CARASAL [101,102]. Neuropathological findings included a diffuse white matter-and myelin pallor, asymmetric fibrous thickening in small arterioles or astroglyosis. Lacunar changes may also be possible, such as perivascular tissue refraction or axonal loss [30]. 

The French family showed linkage with an 11.2-Mb interval on chromosome 20q13, encompassing the 1,145-kb region of the *CTSA* variant, presenting a strong argument that it was the same disease [102]. The *CTSA* gene encodes protective protein cathepsin A (PPCA, genomic coordinates (GRCh37): 20:44 519 590–44 527 458) protein and is located on chromosome 20q13. Structurally, the *CTSA* gene overlaps at its 5′ and 3′ ends, and two other genes were both transcribed from the antisense strand relative to *CTSA*. The gene at the 3′ end (*PLTP*) encoded a phospholipid transfer protein, whereas that at the 5′ end (Neuralized E3 Ubiquitin Protein Ligase 2, *NEURL2*) encodes OZZ, a striated muscle-specific E3-ubiquitin ligase. Thus, mutation analysis of the patient’s *CTSA* gene and biochemical assessment of the cathepsin A, β-galactosidase, and neuraminidase-1 activities in cultured fibroblasts should be performed to support the diagnosis of early infantile galactosialidosis. Recessive *CTSA* mutations may cause galactosialidosis. One of the numerous functions of cathepsin A was to degrade endothelin-1. Thus far, 19 disease-causing mutations have been identified for galactosialidosis in the *CTSA* gene [103], the majority of which were missense mutations resulting in single amino acid substitutions. An incomplete PPCA functional protein was resulted from the absence of *CTSA* mRNA, leading to either a premature termination of translated protein or a truncated protein product [30]. This defective protein was caused by small deletions/insertions, missense mutations, splicing variants, or a nonsense mutation. In addition to cerebral vascular abnormalities, endothelin-1 may have a role in the pathogenesis of CARASAL [30,102].

While homozygous *CTSA* mutations may play a role in galactosialidosis because of a deficiency in β-galactosidase and neuraminidase-1 [103,104], heterozygous *CTSA* mutations were not associated with the disease previously. Although the possible functional role of the *CTSA* mutation in CARASAL remained unexplained, the Arg325Cys missense could have a dominant inheritance and possibly some toxic effect [30]. The extra cysteine could affect the stabilization, folding, and structure of the protein, probably through the extra disulfide bond [74]. Notably, CADASIL was the most common monogenic SVD and is primarily caused by *NOTCH3* mutations that affected cysteines, as described above. On the other hand, a recent report demonstrated that endothelin-1 mediated the inhibition of oligodendrocyte maturation and remyelination by reactive astrocytes [105]. A higher abundance of astrocytic endothelin-1 was detected in the brains of patients with CARASAL compared to controls, potentially leading to reduced cathepsin an activity [30].

CARASAL should be classified as a very rare disease, since currently, limited diagnostic tools were available [30,102]. CARASAL may represent a new phenotype to the spectrum of SVD. More genetic studies on *CTSA* mutations should be performed to assess the prevalence of the disease [30]. Nevertheless, for SVD patients with a positive family history, an unusual extensive leukoencephalopathy, and an absence of *NOTCH3, HTRA1*, and *COL4A1/A2* mutations, a molecular analysis of the *CTSA* gene should be considered [30]. It was worthwhile to investigate whether heterozygous *CTSA* mutations were associated with an increased risk of SVD, particularly in patients with galactosialidosis [30,102]. Further studies should elucidate whether the variant in *CTSA* was associated with CARASAL and whether the risk of an SVD was also seen with other mutations. 

## 5. Hereditary Diffuse Leukoencephalopathy with Spheroids (HDLS)

Hereditary diffuse leukoencephalopathy with spheroids (HDLS) is an autosomal-dominant white-matter disease with a high age-dependent penetrance that differs between genders [106]. The disease is characterized by different clinical symptoms, including dementia and personality changes (depression, schizophrenia, anxiety or irritability) [106,107]. Motor dysfunctions also appear, such as gait instability, pyramidal signs, and an instable posture [31,107,108,109]. Urinary incontinence could also be prominent among the patients [106]. HDLS causes adult-onset cognitive impairment when patients were in their 40s and 50s [31]. Cognitive impairment was the most frequent initial symptom in women whose disease began when they were 20–30 years old [107]. Since the clinical presentation of HDLS has not been clearly elucidated yet, patients may be clinically misdiagnosed as having frontotemporal dementia, multiple sclerosis (MS), cerebral autosomal-dominant arteriopathy with subcortical infarcts, leukoencephalopathy [25], AD [80], or corticobasal degeneration [106]. The disease is characterized by the degeneration of white matter, loss of the myelin sheath, and destruction of the spheroids in axons [108]. MRI findings on patients with HDLS often revealed cerebral white matter lesions, which were asymmetrical in the early disease stage, but become symmetrical with disease progression. Several brain areas were affected, particularly the frontal and parietal white matter [31]. Microbleeds may be missing in cases of HDLS, and grey matter could be spared from disease pathology [109]. 

Colony-stimulating factor 1 receptor [*CSF1R*, MIM*164770] mutations cause HDLS, which was first identified by Axelsson et al. in 1984 [31,110]. As a transmembrane protein, CSF1R served as a tyrosine kinase and was involved in the activation of mononuclear phagocytic cells, such as microglia. Microglia cells participated as immune effector cells and played a role homeostasis and surveillance in the brain [111]. Dysfunctions in microglia due to *CSF1R* mutations was assumed to be the primary disease-causing mechanism in HDLS [112]. On the basis of these observations, the name of adult-onset leukoencephalopathy with axonal spheroids and pigmented glia (ALSP) was proposed to encompass both of these *CSF1R*-related diseases [113]. Most of the mutations were located in the tyrosine kinase domain of CSF1R and were thought to cause CSF1R loss-of-function [31,107]. 

Recently, mutations in *CSF1R* have also been identified in families with pigmented orthochromatic leukodystrophy [113], which was another disease that affects the white matter and was clinically and pathologically similar to HDLS. In addition, functional studies have suggested that the mutations affect the kinase activity of the protein, probably by altering the phosphorylation of downstream targets [31]. Although mutations in the *CSF1R* gene may cause HDLS, mutations in this gene were detected in cases with different clinical manifestations, further demonstrating the difficulties in the clinical diagnosis of HDLS. It was found that 79% of the mutations were located in the distal part of the TKD of CSF1R (102 cases) protein [103]. TKD is a type III receptor tyrosine kinase belonging to the platelet-derived growth factor (PDGF) receptor family, whose members included PDGF-α and PDGF-β, the FMS-like tyrosine kinase 3 (FLT3), and the receptor for stem cell factor (c-KIT). The protein kinase domains, which were a key regulator of most of the cellular pathways that might associate with disease, were structurally conserved and often oncogenic.

**Figure 3:**
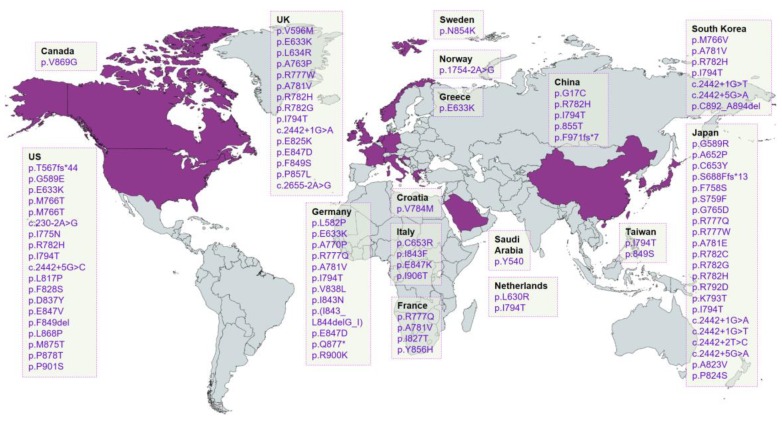
Worldwide distribution of CSF1R mutations. The countries in which the mutation was reported were shown in purple. This world map was created using mapchart (http://mapchart.net/).

The number of HDLS cases confirmed by genetic analysis is increasing in various populations, suggesting that the prevalence of HDLS is higher than previously thought. *CSF1R* mutations were reported in families from the USA, Europe, and Asia—however, there was no obvious relationship between the specific mutations and country or race [107]. Although ALSP was usually inherited in an autosomal-dominant manner, sporadic form of diseases could comprise 40% of the cases in all families [107]. Figure 3 summarized all mutations discovered in the *CSF1R* gene from worldwide. The majority of the described variants were missense mutations. To date, approximately 60 pathogenic variants have been reported in patients with HDLS, including 51 missense-, nine splice-site, two frameshift-, one nonsense mutation, and one deletion mutation. All of these mutations were located within the TKD, however, the p.Thr567fsX44 mutation was found outside of the TKD. Interestingly, the p.Ser688-GlufsX13 mutation was within the kinase insertion domain and could interrupt the TKD [114]. Mutations were found more frequently in the distal kinase domain encoded by exons 17–21 (46 mutations) than in the proximal kinase domain encoded by exons 12–15 (13 mutations). The most common mutation was p.Ile794Thr mutation, which was found in 14 families around the world—a majority of them were found in Japan (8), but additional families were also positive for this mutation from USA (two), Germany (two), Netherlands (two), and Taiwan (two) [107].

A recent study analyzed 122 cases from 90 families with *CSF1R* mutations and demonstrated that the phenotype of HDLS caused by *CSF1R* mutations was influenced by gender. The mean age of onset was 43 years (range, 18–78 years), the mean age at death was 53 years (range, 23–84 years), and the mean disease duration was 6.8 years (range, 1–29 years) [107]. The *CSF1R* mutations were effectively gain-of-function mutations, producing dominant negative repressors [31]. HDLS is an autosomal-dominant disease, and its diagnosis should be essential even in the absence of a positive family history in which a specific *CSF1R* mutation showed a reduced penetrance. Since pharmacological targeting of *CSF1R* with tyrosine kinase inhibitors prevented the disease progression in mouse models of neurodegenerative disorders [2,3,24], a potential pharmacological benefit of CSF1R inhibition remains to be elucidated for patients with HDLS [115]. Since mutations in the *CSF1R* gene could cause HDLS, further genetic studies should be undertaken to address the difficulties in the clinical diagnosis of HDLS. Probably, these aforementioned facts about the role of *CSF1R* in monocyte/macrophages, as well as in microglia biology, suggested a fundamental impact and therapeutic potential for HDLS.

## 6. COL4A1/2-Related Brain Small-Vessel Disease

Collagen type IV alpha 1 chain (*COL4A1*) related disorders were characterized as cerebral SVD with diverse disease phenotypes that include porencephaly, seizures, dementia, intellectual disability, migraine, stroke, visual impairment (visual loss, cataract, glaucoma), muscle dysfunction or hereditary angiopathy with nephropathy, aneurysms, and muscle cramps (HANAC) syndrome [32,116]. The disease phenotypes appear in early lifetime, even in childhood, but also in the adult life. Imaging of *COL4A1*-related disease revealed changes in white matter, porencephalic cyst, haemmorrhage-or microhaemorrhage, possible involvement in basal ganglia [117,118]. Collagen type IV alpha 2 chain (*COL4A2*) related disease was also suggested as an adult onset SVD, where similar phenotypes were present, such as porencephaly, scattered white matter lesions, carotid aneurysm, myopia, amblyopia, cerebellar- and visual abnormalities [119]. *COL4A1* and *COL4A2* encoded the collagen chains α1 (IV) and α2(IV), respectively, which constituted a major component of the vascular basement membrane [120]. *COL4A1* and *COL4A2* mutations have been reported in a broad spectrum of disorders, including myopathy, glaucoma, cerebrovascular disease, and renal, ophthalmological, cardiac, and muscular abnormalities, which were collectively termed “*COL4A1* and *COL4A2* mutation-related disorders” [32,121,122,123,124]. Furthermore, missense mutations in COL4A1/COL4A2 caused rare familial forms of cerebral SVD, manifesting as deep intracerebral hemorrhages, lacunar ischemic strokes, and WMHs [35,118]. These mutations were associated with porencephaly and infantile hemiparesis and have been recently recognized as a monogenic cause of SVD that could present in adulthood.

Collagen type IV alpha 1 chain *(COL4A1*; NM_001845) and Collagen type IV alpha 2 chain (*COL4A2*, NM_001846) genes comprised of 52 and 48 exons, respectively, and were arranged head-to-head on opposite strands of human chromosome 13 (13q34). *COL4A1* and *COL4A2* genes, located on chromosome 13q34 (in tandem), shared a unique, bidirectional promoter. Their transcripts were strongly controlled by epigenetic mechanisms, including regulation by a microRNA family, which could be involved in the down-regulation of their expression [83,84]. Additional microRNAs may also indirectly regulate collagen synthesis [125,126]. Three main domains of COL4A1 and COL4A2 proteins could be distinguished: an N-termimal 7S domain, a central triple-helix-forming (collagenous) domain, and a C-terminal non-collagenous (NC1) domain. Human and mouse *COL4A1* were similar, since they both had 21 conservative repeats, which divided the collagenous domain into 22 sub-domains. In addition, they both had 23 conserved repeat interruptions which align with those in *COL4A1*. All cysteine residues in the collagenous domain of *COL4A1* and *COL4A2* were present inside repeat interruptions, which suggested that these cysteine repeats may also be important sites for cross-linking between different molecules [123]. There were multisystem disorders that result from *COL4A1* and *COL4A2* mutations. Emerging studies were available on *COL4A1* mutations, and the first *COL4A2* mutations were recently reported [127,128]. In total, data from 67 families with *COL4A1* and *COL4A2* mutations were reported [124]. In these families, 33 mutations (50%) were found associated with familial form of disease—a de novo mutation was identified in 17 cases (25%), and in 17 cases (25%), there was a lack of data on parental sequencing [124]. These data indicated that the de novo mutation be *COL4A1* and *COL4A2* was high. In addition, with a high de novo mutation rate of 40%, 21 *COL4A1* (12 of them novel) and 3 *COL4A2* pathogenic mutations were identified, mostly in children with porencephaly or other patterns of parenchymal hemorrhage [124]. 

Together, these findings suggested that patients with *COL4A1* and *COL4A2* mutations may be at a higher risk of retinal hemorrhages, and retinal examinations may be useful for identifying patients with *COL4A1* and *COL4A2* mutations who may also be at a higher risk of hemorrhagic strokes [129]. In addition to prenatal and perinatal hemorrhages, *COL4A1* and *COL4A2* mutations also caused the sporadic and recurrent intracerebral hemorrhages (ICH) in young and old patients [123]. Most of the reported *COL4A1* mutations were heterozygous missense mutations that affected a glycine residue within the collagenous region of the protein. However, many studies have shown that dominant missense mutations in *COL4A1/COL4A2* caused rare familial forms of cerebral SVD, manifesting as deep ICHs, lacunar ischemic strokes, and WMHs [35,118]. Genes causing rare familial forms of cerebral SVD may also contain variants conferring risk for common forms of cerebral SVD [130,131].

The *COL4A1* and *COL4A2* mutations were important causes of cerebrovascular disease with a high mutation detection rate in porencephaly and childhood cerebral hemorrhage, and a relatively high rate of de novo mutations. This mutation was less prominent in (sporadic) adult-onset intracerebral hemorrhage, with an incidence of 6% [127,132]. In addition, the precise role of *COL4A1* and *COL4A2* mutations in cortical malformations needed to be elucidated, but it appeared to contribute to the malformations that resulted from vascular insults during fetal development [133]. Follow-up data on *COL4A1* and *COL4A2* mutation carriers would be important for developing appropriate surveillance protocols and adapting treatment.

## 7. Fabry Disease

Fabry disease, an X-linked lysosomal storage disorder, is caused by the deficiency or absence of alpha-galactosidase A (α-Gal-A), which leads to an accumulation of globotriaosylceramide/ceramide trihexoside (Gb3) with a terminal a-D-galactosyl residue, particularly globotriaosylceramide (GL-3, Gb3, CTH) and globotriaosylsphingosine (Lyso-GL-3, lyso-Gb3) [134,135]. This enzyme deficiency results in the accumulation of globotriaosylceramide within the lysosomes of various organs, such as the blood vessels, kidneys, heart, and dorsal root ganglia. The main disease symptom is acroparaesthesia, which appears in childhood or early adulthood. Many different clinical phenotypes were observed in patients with Fabry disease such as corneal dystrophy, angiokeratomas, visual impairment, reduced cognitive function, hearing loss, dizziness, problems with digestion, vertigo, depression, or abnormalities in cardiovascular system [135]. Patients with classical Fabry disease could represent the neuropathic pain, verticillata, and angiokeratoma, followed by stroke, heart disturbances, and kidney failure. The atypical form of the disorder has a later disease onset. The disease progression may affect a single organ or have a milder phenotype [136]. The most prominent MRI finding in Fabry disease was multifocal leukoencephalopathy, but additional abnormalities may be also observed, such as lacunar ischaemic lesions (basal ganglia, brainstem), cerebral atrophy or pulvinar sign [137]. The disease affected both myelinated and unmyelinated neurons, resulting in symmetrical small fiber neuropathy. It started from the feet and spreads proximally. Both males and females are affected, but the disease phenotype is more severe and has an earlier onset in affected males than in females [138]. 

Fabry disease is caused by the genetic mutations in the alpha-galactosidase-A gene (*GLA*-gene), located on the long arm of the X-chromosome (Xq22.1). Thus far, more than 790 different mutations have been described, including missense and nonsense mutations; however, small deletions, insertions, and splicing defects have also been reported in “The Human Gene Mutation Database” (http://www.hgmd.cf.ac.uk/ac/gene.php?gene=GLA, accessed on 1 July 2019). Most of the pathogenic *GLA* mutations were restricted to single families [139]. Interestingly, the disease manifestations may vary within families carrying the same mutation [140], however, the genotype-phenotype correlation was confined to individual families. In general, however, mutations that have been associated with more attenuated late-onset disease were frequently missense mutations, e.g., the N215S genotype. One missense variant, the D313Y genotype, was considered to be a non-damaging polymorphism [141]. The incidence of Fabry disease has been estimated to be 4.5% in men and 3.4% in women among patients with cryptogenic stroke [142], whereas the incidence was one per 17,000 to 117,000 in the general population [143,144]. The prevalence ranges from 0.6% to 11.1%. In addition, the disease seems to be rare, with an estimated prevalence of one in 40,000 to 60,000 in males of all ethnicities. As an X-linked disease, the genetic defect that causes Fabry disease could be transmitted by both males and females, but it seemed to be transmitted less frequently in females. Remarkably, the only coding variant (c.937G > T, p.Asp313Tyr) was detected approximately ten times more frequently (approximately 5%) in our cohort than in the previous study, which reported a frequency of 0.45% [145].

Many missense mutations affecting cysteine residues were associated with the classic Fabry disease phenotype due to the formation of disulfide bonds in the wild-type α-Gal structure, resulting in negligible or reduced residual enzyme activity [146]. This observation attests to the importance of these genes for normal enzyme function. Several missense mutations that introduced an extra cysteine into the amino acid sequence of α-Gal have been identified in Fabry disease patients. The Cys174Arg [115,147] mutation was expected to be a pathogenic mutation. *GLA* Cys174Gly were recently identified in a patient presenting with an unusual late-onset renal variant of Fabry disease [148], suggesting that this mutation was not clinically benign [149]. In addition, the *GLA* Arg118Cys variant was recently reported in large case-finding studies in different European populations [150,151,152,153] and Brazil [154] among patients with stroke, left ventricular hypertrophy, or on chronic dialysis. However, the *GLA* variants that cause Fabry disease phenotypes were still inconsistent. More recently, Ferreira et al. (2015) reported that the *GLA* Arg118Cys caused the Fabry disease phenotype; the findings were based on the analysis of a series of Iberian (Portuguese and Spanish) individuals and families [146]. Surprisingly, carriers of the Cys118 allele may present only with a typical eruption of angiokeratomas, which was usually considered a manifestation of classic Fabry disease. These data may explain the absence of a familial history of Fabry disease in an Italian patient identified by newborn screening [155,156]. The identification of a *GLA* mutation associated with a clinically relevant phenotype was crucial for patient management and risk stratification. However, the unresolved issue of how to appropriately match complementary genotypes and phenotypes remains a major impediment to the clinical application of such diagnostic information.

## 8. Possible Mechanisms of Cerebral Small Vessel Diseases Association Genetics

Several genes and mutations were identified in familial SVD and described above. These variants in phenotype share both clinical and radiological features with sporadic SVD and provide important insights into the mechanisms of the disease. SVD were known as complex disorders [1,22,42]. SVD disorders have several subtypes (such as CARASIL, CADASIL, HDLS, Fabry disease), and the exact molecular mechanisms of these diseases remain incompletely understood [1,22,40,42]. Several risk factors that could play a role in SVD have been identified, such as aging, head injury or stroke. Possible mechanisms of dysfunctions could be associated with dysfunctions in vessels, reduced defensive mechanism, and disturbances in vascular reactivity [42]. Post-mortem and animal studies were performed to find out the exact pathways, associated with SVD mechanisms [22,157]. The classical hypothesis of disease suggests that it was caused by cerebral reduced blood flow and the impairment of cerebral autoregulation. Elevated permeability and disruption of the blood-brain barrier (BBB) may also play a significant role in disease [20]. Reactive oxygen species (ROS) were also suggested to be important contributor in SVD. NADPH oxidases serve as ROS producing enzymes and could play a role in vascular diseases by producing superoxides [34]. In addition, genetic mutations in different genes could also play a role in the onset of different forms of SVD. Dysfunction in the extracellular matrix pathways, were associated with different diseases, such disturbances in potassium channels by NOTCH3 aggregation (CADASIL). NOTCH3 is a single transmembrane receptor involved in the Notch signaling. Mutations resulted in dysfunctions in the Notch signaling pathways. Mutant NOTCH3 accumulated in the vasculature and formed granular osmiophilic material (GOM). The exact role of GOMs in CADASIL remained unclear, but it may be possible that they result in impairment in glymphatic system. NOTCH3 also played a role in the development and homeostasis vascular system, and its dysfunctions could result in abnormal small vessel development, breakdown of BBB [47]. In addition, the genetic pathogenesis similarities existed between CADASIL and Alzheimer’s disease, affecting the small vessels of the brain. Depending on the mutation, plausible dual molecular mechanisms could be involved in vascular damage and their impact on brain function, meaning one gene could influence both diseases [2,23,24,158,159,160].

HTRA1 gene encodes a serine protease and is involved in TGFβ signaling and angiogenesis. Mutations in HTRA1 cause CARASIL by the attenuation of TGFβ function. HTRA1 cleaves the latent TGF-β binding protein 1 (LTBP-1), which is an important regulator of TGF-β [161]. *COL4A1/COL4A2* related angiopathy was associated with collagen dysfunctions and ER stress, but the exact mechanism remains unclear. Apoptosis could play an important role in the onset of microcephaly [22]. *CSF1R* is a tyrosine kinase involved in HDLS which plays a key role in development and survival of microglia. Mutations in the receptor domain of CSF1R eliminated the kinase activity, ability of dimer formation, and CSF1-CSF1R interaction. CSF1R deficiency resulted in a reduction of microglia density and neuronal loss [31]. *CTSA* mutations (CARASAL) caused in endothelin-1 degradation and reduced oligodendrocyte regulation [30]. Additional pathways could also be associated with SVD causing mutations, for example, they could impair the blood-brain barrier integrity or the protection against DNA damage [22]. CSF1R is a tyrosine kinase transmembrane protein involved in activation of mononuclear phagocytic cells, for example, microglia that function as immune effector cells with homeostatic and surveillance tasks in the brain [111]. As microglial dysfunction due to *CSF1R* mutations is assumed to be the primary disease-causing mechanism, HDLS is classified as microgliopathy [111].

In several cases, genetics of SVD remained unexplained. Genome-wide association studies (GWAS) discovered several possible genetic factors that could contribute to sporadic form of SVD, such as *NEURL1* (E3 ubiquitin protein ligase 1), *PDCD11* (programmed cell death 11) and *SH3PXD2A* (SH3 and PX Domains 2A), which could be related to WMHs. Currently, it remains unclear whether the disease associated pathways were similar in all individuals, or whether there was additional different disease mechanism. GWAS revealed SVD-related pathways, but in some cases, these pathways were shared [22]. Heterozygous frameshift mutations in *TREX1* (three prime repair exonuclease 1) were also be involved in vascular dysfunctions, and caused cerebroretinal vasculopathy or hereditary endotheliopathy, retinopathy, nephropathy and stroke. These mutations cause premature STOP codon and could possibly reduce the DNA repair mechanisms in case of oxidative stress. This process could result in premature aging in the vascular system [162]. *FOXC1* (forkhead box transcription factor) duplications or deletions could cause abnormal development of cerebellum, impaired neovascularization in cornea. Missense mutations may also affect the *FOXC1* protein function. Brain MRI of patients with *FOXC*1 mutations (duplication and deletion) revealed WMHs, lacunar infractions and dilated perivascular spaces. *FOXC1* dysfunctions may impair the vascular cell stability by inducing dysfunctions in development of neural crest and mural cell recruitment. *PITX2* (paired like homeodomain 2) could interact with *FOXC1*, and associated with several dysfunctions, such as atrial fibrillation, cardioembolic stroke, and white matter hyperintensities in brain [163]. 

Although the roles of most of the common genetic variants (e.g., *NOTCH3*, *HTRA1*, *CTSA*, *CSF1R COL4A1*, *COL4A2*, and *GLA*) emerging in association with sporadic forms of cerebral SVD are yet to be fully elucidated, it became clear that each of these variants could potentially disrupt specific components of the neurovascular unit’s structure and function. A detailed characterization of these pathways would be needed for the discovery of novel molecular targets for future therapies. Shared pathways affecting the integrity and function of the extracellular matrix appeared to play an integral role in these disease’s pathways. It was likely that there were multiple shared pathways, each being involved to different degrees in different manifestations or subtypes of SVD. These genetic mechanisms, as well as their interactions with environmental factors, may provide explanations as to why different patients in the sporadic disease population exhibit each feature of SVD to a different extent.

## 9. Conclusions and Future Perspective

Studies in both monogenic forms of SVD and the genetics of sporadic SVD were able to fill in the blank edges in the map of the disease processes in SVD. Blood circulation in the brain may be impaired by conditions that affect the large vessels, causing ischemic and hemorrhagic infarcts, and the small vessels, causing cerebral SVD. The pathological processes of cerebral SVD are complicated and incompletely understood. Hereditary SVDs were associated with cognitive and motor impairments, as well as stroke. These diseases affected not only the brain, but other organs as well, such as the kidney or visual system. Currently, no effective therapy is available for SVD [164]. In the last few years, GWAS and next generation sequencing techniques have facilitated the discovery of genes which could be involved in SVDs [1]. Genetic studies on the various forms of SVD could reveal a shared pathogenesis, re-conceptualizing the development and progression of cerebral SVD and closing the gaps in our understanding of the disease processes. These genetic mechanisms, as well as their interactions with environmental factors, may provide explanations and support for diagnosing the various forms of cerebral SVD. Since a correct diagnosis may be difficult to achieve based solely upon the clinical symptoms, molecular testing is becoming more widely available [165]. Patients should be more frequently diagnosed, and a clearer picture will emerge of both the prevalence and clinical spectrum of the disease. 

Hence, the genetic factors of cerebral SVD play a pivotal role in terms of unraveling their molecular mechanism, and further studies could be considered a powerful tool for understanding the disease. Further genetic studies in cerebral SVD will likely provide further insight into the mechanisms involved in both the monogenic and sporadic forms of the disease. If large enough patient cohorts were available, a whole-genome association study could be an effective way to study the genetic factors underlying the phenotypes. Thus, further studies of autosomal-dominant diseases could use linkage analysis paired with targeted re-sequencing or whole-genome sequencing. Together, these studies could aid in the development of novel diagnostic strategies and treatments for the various forms of cerebral SVD. A challenge in genetic studies could be the lack of cell and animal models, which may be helpful in understanding the disease mechanism in terms of mutations. Studies on epigenetics and gene environmental interactions may also open more avenues in the prediction of risk for SVD [21]. In addition, some important issues with cerebral SVD were clinically heterogeneous and share several common features with other neurodegenerative diseases [43], such as a possible genetic background, assembly of misfolded proteins, or neuronal loss rather than a focal brain disease [166,167,168]. Thus, maximizing information gained from genetic studies requires collaborative study utilizing thousands of human samples as well as cell culture and animal models to clarify specific disease mechanisms. Once disease mechanisms underlying cerebral SVD are established, individual genetic risk profiles may suggest particularly effective medical or dietary therapies to halt cerebral SVD’s common and devastating manifestations. In addition, despite intensive research efforts, the molecular mechanisms underlying cerebral SVD remain poorly understood, which has hampered the development of cerebral SVD-specific therapies. Important steps forward in understanding the disease mechanisms underlying cerebral SVD have been made using genetic approaches in studies of both monogenic and sporadic SVD. Further genetic studies in SVD will likely provide more conclusive evidence of an overlap of disease pathways involved in both monogenic and sporadic disease. Although understanding the processes of each disease, whether a monogenic form of SVD or sporadic disease, may aid the development of treatment options for the specific disease, it is possible that the distinction between each of the diseases is blurred, and that the same few convergent processes will eventually serve as therapeutic targets.

## Figures and Tables

**Table 1:** Characteristics of single-gene disorders causing cerebral SVD.

Diseases	Inheritance	Chromosome Locus	Gene	Mutation Findings	Protein	Cerebral Features	Neuroimaging Finding	Pathological Findings	References
**CADASIL**	Autosomal dominant	19q12	*NOTCH3*	Over 256 missense mutations or rare deletions and insertions were reported	Transmembrane receptor	Migraine with & aura, recurrent was chemic strokes, mood disturbance, cognitive decline, disability > death 65–70 years WMH, lacunar infarcts, dilated PVS, micro bleeds, brain atrophy	Typical of cerebral SVD plus in anterior temporal lobe and external capsular	Granular osmiophilic material found in the walls of affected arterioles	[27]
**CARASIL**	Autosomal recessive	10q25	*HTRA1*	At least 17 mutations were identified in 25 families worldwide	HtrA serine peptidase/protease 1	Recurrent is chemic strokes, Cognitive decline, disability > death	Similar to CADASIL, diffuse WM lesions and small infarcts in basal ganglia	Arteriosclerotic changes, WM changes. No GOM deposition. Hyaline degeneration and thickening and splitting of internal lamina	[28,29]
**CARASAL**	Autosomal dominant	20q13.12	*CTSA*	Many galactosialidosis patients related to *CTSA* gene point mutations were reported	Cathepsin A (CathA)	Therapy-resistant hypertension, strokes, and slow and late cognitive deterioration	A diffuse, progressive leukoencephalopathy preceding the onset of strokes and disproportionate to the degree of clinical severity	Endothelin-1 overexpression coincides with increased numbers of premyelinating OPCs, decreased MBP amounts, abundance of axons without myelin, and features of remyelination failure	[30]
**HDLS**	Autosomal dominant	5q32	*CSF1R*	Approximately 60 pathogenic variants have been reported in patients with HDLS	CSF-1 receptor	Stroke episodes with pyramidal, bulbar and	Diffuse WM lesions, lacunar strokes and atrophy	Diffuse gliosis, moderate loss of axons and many axonal spheroids	[31]
**COL4-related disorders**	Autosomal dominant	13q34	*COL4A*, *COL4A2*	Over 50 types of mutations have been reported to dat	Collagen type IV, alpha chains	Infantileemiparesis, intracerebralhemorrhage (perinatal, youngoradult) porencephaliccysts, microbleeds, WMH, intracranial aneurysms (HANAC)	Typical of cerebral SVD	Defects in the basement membrane	[32]
**FD**	X-linked	Xq22	*GLA*	Around 585 pathogenic mutations have been reported in the *GLA* gene to date	Lysosomal α-galactosidase A	Typically, stroke is considered a manifestation of end stage	Multifocal WMH lesions, intracranial arterial dolichectasia	Lysomal storage materials in vascular endothelial cells and smooth muscle cells	[33,34]

CADASIL: Cerebral autosomal dominant arteriopathy with subcortical infarcts and leukoencephalopathy; CARASIL: Cerebral autosomal recessive arteriopathy with subcortical infarcts and leukoencephalopathy; AD: autosomal dominant; AR, autosomal recessive; SVD: small vessel disease; WMH: white matter hyperintensities; HDLS: Hereditary Diffuse Leukoencephalopathy with Spheroids; RVCL: retinal vasculopathy with cerebral leukodystrophy; FD: Fabry disease; OPC: Oligodendrocyteprogenitor cells; MBP: myelin basic protein.

**Table 2:** Various typical clinical CADASIL-causing variants in *NOTCH3* that do not affect cysteine amino acids.

Mutation	Exon	Age of Onset	Gender	Population	Clinical Phenotypes	References
p.Arg61Trp	2	46	NA	American	Migraine, aphasia, hemiparesis, probable familial	[66]
p.Arg75Pro	3	53	M	Korean	Typical CADASIL symptoms, granular osmiophilic granules on skin biopsy in a patient. Probable positive family history	[67]
p.Arg75Pro		47	F			
p.Arg75Pro		65	M			
p.Arg75Pro		40–50s	F	Japanese	Proband: depression, akinetic mutism, pseudobalbar palsy, and tetraplegia; MRI: mild frontal and temporal lobe atrophy multiple cerebral infarctions at the bilateral basal ganglia other family members: stiffness, headache, vertigo, dizziness; MRI: e infarctions at the bilateral basal ganglia	[80]
p.Arg75Pro		64	F		right thalamic hemorrhage, akinetic mutism, pseudobalbar palsy, repeated cerebral infarctions MRI: diffuse cerebral atrophy Family members were affected with dementia	
p.Arg75Pro		34	M	Chinese	Cerebral infarction, transient ischaemic attack with tinnitus, irritable; MRI: small infract in different brain areas (deep white matter, basal ganglia)	[79]
p.Asp80Gly		50s	F	German	Imaging and clinical symptoms: typical CADASIL phenotype. Mutation may be segregated with diseases	[54]
p.Arg107Trp		NA	NA	German	CADASIL, no clinical phenotype	[68]
		63	M	UK	Hypertension, Mother was affected with stroke	[84]
p.Pro109Thr		57	F	Iranian	CADASIL, no detailed clinical phenotype. Mutation co-existed with NOTCH3 Pro203His mutation.	[78]
p.Gly149Val	4	39	F	Chinese	Initial symptoms: progressive dizziness, memory dysfunctions. MRI: leukoencephalopathy and confluent signal abnormalities; Family history probable positive	[69]
p.Gln151Glu		NA	NA	Italian	CADASIL, white matter hyperintensities, migraine, no detailed clinical phenotype	[68]
p.Gln151Glu		NA	NA	Spanish	CADASIL, white matter hyperintensities, stroke, no detailed clinical phenotype	[70]
p.His170Arg		NA	NA	Spanish	CADASIL, white matter hyperintensities not specified, no detailed clinical phenotype	[70]
p.His170Arg		NA	NA	Oceanian	CADASIL, not specified white matter hyperintensities, stroke, no clinical phenotype	[71]
p.Ala198Thr		NA	NA	Italian	CADASIL, white matter hyperintensities, migraine, no detailed clinical phenotype	[68]
p.Ala202Val		NA	F	Oceanian	CADASIL, not specified white matter hyperintensities, stroke, no clinical phenotype	[70]
p.Pro203His		57	F	Iranian	CADASIL, no detailed clinical phenotype. Mutation co-existed with NOTCH3 Pro109Thr mutation.	[78]
p.Arg207His		NA	NA	Italian	CADASIL, white matter hyperintensities, migraine no detailed clinical phenotype	[68]
p.Arg213Lys		36	M	Japanese	CADASIL, white matter hyperintensities, migraine, dementia, stroke, positive family history	[71]
p.Arg213Lys		10	M	Japanese	CADASIL, white matter hyperintensities, dementia migraine, stroke, positive family history	[72]
p.Val237Met	5	71	F	Japanese	CADASIL, white matter hyperintensities, stroke, gait disturbances, dementia, positive family history	[81]
p.Val252Met		63	M	Russian	strokes and/or transient ischemic attacks, pseudobulbar palsy, pyramidal signs. MRI: hyperintensities of the external capsules, temporal lobes, lacunar infarcts in the cerebellum and/or brainstem, cerebral hemispheres	[73]
p.Glu309Lys	6	NA	NA	Italian	CADASIL, white matter hyperintensities, migraine, dementia, family history positive	[68]
p.Ser497Lys	9	NA	NA	Russian	CADASIL, non-specified white matter hyperintensities, appeared in unaffected individuals	[73]
p.Thr577Ala	11	NA	NA	Portuguese	Unclear phenotype, non-specified white matter hyperintensities	[74]
p.Arg592Ser	11	NA	NA	Italian	CADASIL, white matter hyperintensities, migraine, dementia, probable positive family history	[68]
p.Val644Asp	12	NA	NA	Italian	CADASIL, white matter hyperintensities, migraine, dementia, probable positive family history	[68]
p.Ser978Arg	18	NA	F	Portuguese	CADASIL, white matter hyperintensities, stroke seizure, psychiatric symptoms	[74]
p.Ala1020Pro	19	Adolescence	F	German	CADASIL, white matter hyperintensities, hypertension, migraine, dementia, positive family history	[82]
p.Ala1020Pro	19	NA	F	German	CADASIL, white matter hyperintensities, hypertension, psychiatric disturbance, positive family history	
p.Thr1098Ser	20	39	M	Chinese	CADASIL, white matter hyperintensities, stroke, psychiatric disturbances, dementia, positive family history	[79]
p.His1133Gln	21	Unknown	Unknown	Russian	CADASIL, non-specified white matter hyperintensities,	[85]
p.His1235Lys	22	Unknown	Unknown	Italian	CADASIL, white matter hyperintensities	[68]
p.Lys1515Pro	25	35	F	French	CADASIL, white matter hyperintensities, migraine, dementia, positive family history	[75]
p.Val1762Met	29	Childhood	F	Italian	CADASIL, white matter hyperintensities, psychiatric dysfunctions, positive family history	[77]

**Table 3:** *HTRA1* mutations in patients with CARASIL.

Mutation	Exon	Gender	AOO	Clinical Phenotypes	Mutation Type	Ethnicity	References
*Asian patients*							
p.Ala252Thr	3	F	Teenage	Lumbago in her teens, leukoaraiosis in brain, stroke, gait disturbance, pseudobulbar palsy, pyramidal signs	Homozygous missense	Japanese	[28]
p.Val297Met	4	M/F	14–16	Alopecia in teens, leukoaraiosis in brain, spondylosis, dementia, gait disturbance, pseudobulbar palsy, pyramidal signs			
p.Arg302X	4	F/M	14/16	Alopecia in teens, spondylosis, dementia, gait disturbance, possible stroke, pseudobulbar palsy, pyramidal signs			
p.Arg370X	6	F	18	Alopecia in teens, spondylosis, dementia, gait disturbance, possible stroke, pseudobulbar palsy, pyramidal signs			
p.Arg274Gln	4	F	14	Lumbago in her teens, lumbar and cervical spondylosis in her 30s, later subcortical ischemic lesions and spastic paraparesis and intellectual dysfunctions			[76]
p.Gly283Glu	4	M	49	Cognitive impairment, gait disturbance, spondylosis, pseudobulbar palsy, hyperreflexia in limbs	Heterozygous missense		[99]
p.Pro285Leu	4	M	20	Alopecia in his 20s, gait distubances in his 30s, cognitive dysfunction in his 50s. Spondylosis, hyperreflexia in limbs, Babinski reflexes			
		M	51	Stroke, cognitive impairment and gait disturbance in his 50s. Spondylosis, pseudobulbar palsy, hyperreflexia in limbs			
p.Arg302Gln	4	M	20-63	Alopecia possible in their 20–40s. Spondylosis, pseudobulbar palsy (not all patients), hyperreflexia in limbs, Babinski reflex			
p.Thr319Ile	4	M	53	Spondylosis, pseudobulbar palsy, hyperreflexia in limbs, Babinski reflex			
p.Pro285Leu	4	F	24	Progressive alopecia from birth, right limb disability, lumbar pain, lethargy, memory dysfunctions	Homozygous missense	Chinese	[91]
p.Leu364Pro	6	F	25	right foot and lumbar pain, prolapse of lumbar intervertebral disk, mild alopecia, intellectual dysfunction, spastic gait Babinski signs	Homozygous missense		[92]
p.Gly56Alafs*160	1	M	28	recurrent stroke, hair loss and low back pain, lower limb weakness, alopecia, pyramidal signs	Homozygous/Frameshift		[85]
*Caucasian patients*							
p.Gly295Arg	4	M	34	Alopecia, dysarthria, dysphagia, emotional instability, and spastic gait, Babinski signLater, cognitive impairment and upper limb weakness, pseudobulbar syndrome	Homozygous missense	Spanish	[94]
p.Arg370X	6	F	29	Alopecia, back and neck pain, right-sided weakness, difficulty in walking	Homozygous nonsense	Turkish	[96]
p.Glu42fs	1	F	29	chronic lumbar and cervical pain from the age of 14, ischemic strokes with left hemiparesis and dysarthria, without alopecia and cognitive dysfunctions	compound heterozygous deletion & missense	Romanian	[95]
p.Ala321Thr	4						
p.Arg166Cys	3	M	33	Alopecia, transient ischemic attacks, lacunar strokes, cervical, and lumbar pain, acute psychosis, cognitive impairment, dysarthria, spastic paraparesis, hyperreflexia, bilateral Babinski’s sign.	Homozygous missense	Portuguese	[100]
p.Gly206Arg	3	M	24	Alopecia, chronic back pain presented with recurrent ischemic strokes, hearing impairment	homozygous missense	American	[97]
